# Prevalence and Associated Risk Factors of Childhood Obesity in the Eastern Province of Saudi Arabia

**DOI:** 10.7759/cureus.30015

**Published:** 2022-10-07

**Authors:** Abdullah Almaqhawi, Abdullah Alkhateeb, Arwa K AlHussain, Khuolod S Alqahtani, Abdulrhman K Aldrweesh, Saleh A Aljarri

**Affiliations:** 1 Family and Community Medicine, College of Medicine, King Faisal University, Hofuf, SAU; 2 College of Medicine, King Faisal University, Hofuf, SAU

**Keywords:** overweight, prevalence, risk factors, children, obesity

## Abstract

Introduction

Overweight and obesity are defined as excessive fat accumulation that poses a risk to well-being. In children, a BMI of the 85th percentile to less than the 95th percentile is considered overweight, and the 95th percentile or greater is considered obese.

Aim

This study aimed to measure the prevalence of childhood obesity in a population of six to 16 years of age and its associated risk factors.

Participants and methods

This is a cross-sectional study conducted among children aged between six and 16 years old who live in the Eastern province of Saudi Arabia. The data collector in each school distributed envelopes containing an informed consent form and a survey to collect data on the demographics and socioeconomic status to all students. All students whose parents signed the informed consent would undergo measurement of growth parameters which ultimately determined the BMI.

Results

Six hundred eighty-eight children agreed to participate. The prevalence of overweight and obesity was 15.3% (overweight 10.2%, obese 5.1%). Children who eat four or more meals per day were 29.5% while only 10% were regularly exercising for more than two hours a day. Independent risk factors of obesity and overweight were eating four or more meals per day. Spending more than an hour a day on physical activity was a protective factor against being overweight and obese.

Conclusion

Increased daily food consumption was the most frequent risk factor for obesity. Physical activity was the protective factor against obesity in children and adolescents. Further intervention measures must be implemented to reduce the prevalence of overweight and obesity. A healthy lifestyle based on effective dietary education and physical activity promotion is required to prevent overweight and obesity among youth.

## Introduction

Overweight and obesity are defined as excessive fat accumulation that causes a risk to one’s well-being [1,2]. According to the Weight Status Category (WSC), individuals in the 85th to 95th percentile are considered overweight, whereas those in the 95th percentile or greater are considered obese [3]. Obesity is concerning because overweight and obese children are likely to become overweight and obese adults, which places them at risk of several chronic health issues, including cardiovascular diseases (CVDs), such as stroke, which are one of the leading causes of death worldwide [3]. Additionally, obesity can lead to several other illnesses, including diabetes, musculoskeletal disorders, and some types of cancer [1,3]. Childhood obesity is associated with the premature onset of some diseases with severe complications and increased mortality [1,2]. Several factors can be linked to childhood obesity, most notably a deficiency in energy metabolism during infancy and adolescence [4]. The excess energy is retained as fat because the energy consumed is greater than the energy expended [4]. This typically occurs due to inactive behaviors or a lack of physical activity. The main factors causing children to become obese are lifestyle, environmental, genetic, and medical [4].

Few studies specifically focus on children with obesity. According to the World Health Organization (WHO), the prevalence of obesity increased globally three times between 1975 and 2016 [1]. In 2019, around 38.2 million children under the age of five were overweight or obese. Almost half of these children lived in Asia. Finally, in 2016 there were more than 124 million obese school-aged children in Sharjah, UAE (6% of girls and 8% of boys) [5].

A 2017 Nepal study found that over one-quarter of children were overweight or obese [6]. Another study conducted in Oman in 2018 showed that 17.4% of the country’s children were overweight or obese [7]. In Riyadh, Saudi Arabia, a study conducted in 2015 reported an increasing prevalence of overweight or obese citizens (18.2%) compared to 12.7% in 2002 (according to the WHO) [3].

Several risk factors contribute to obesity. Many studies have reported a correlation between diminished physical activity and obesity [8-10]. In a previous study, there was a significant relationship found between maternal obesity and childhood obesity [11]; however, there was no relation (p < 0.05) between specific dietary habits and obesity among children [12]. Another study found a significant relation between obesity and sedentary lifestyle (p > 0.05) [13]. Also, 87% of members of the middle socioeconomic class were either overweight or obese (p > 0.05) [13]. No association was reported with gender in a study conducted in Riyadh [2].

This paper aims to measure the prevalence of childhood obesity between six and 16 years of age and its associated risk factors, supplementing the data on this topic in the Eastern Province of Saudi Arabia. The results of this study could promote awareness and prevention of obesity and its complications.

## Materials and methods

A cross-sectional population-based study measured the weight, height, and BMI of healthy asymptomatic school-aged Saudi children aged six to 16 years of both sexes, attending primary and intermediate schools in Al‐Ahsa, from December 2021 to March 2022.

All children who were younger than six years old and older than 16 years old were excluded.

The weight and height of the participants were measured in the school by a trained team of medical students. Weight was measured with the students wearing light clothing and no shoes, using an electronic scale to the nearest 100 g. Height was measured using a wall-mounted stadiometer, with the children not wearing shoes. BMI was calculated as the ratio of weight (kg) to the square of height.

The data collector in each school distributed envelopes to all students. Each envelope contained an informed consent form and a survey that collected data on demographics and socioeconomic status.

All students whose parents signed the informed consent went for the measurement of growth parameters. The parental socioeconomic status was measured by collecting data on four main indicators: parents' educational level, family income, habitation, and parents' jobs. The subjects were selected randomly through online questionnaires [2].

Statistical analysis

Descriptives were presented as numbers and percentages (%). BMI was grouped as underweight or normal vs overweight or obese. The association between the BMI level and sociodemographic characteristics of children has been performed using the chi-square test. Based on the significant results, a multivariate regression model was carried out to determine the significant independent factor associated with overweight or obesity with a corresponding odds ratio and a 95% confidence interval. Two-tailed analyses with p<0.05 were used as a statistical significance cutoff. All data analyses were performed using the Statistical Package for Social Sciences, version 26 (SPSS, Armonk, NY: IBM Corp, USA).

## Results

**Figure 1:**
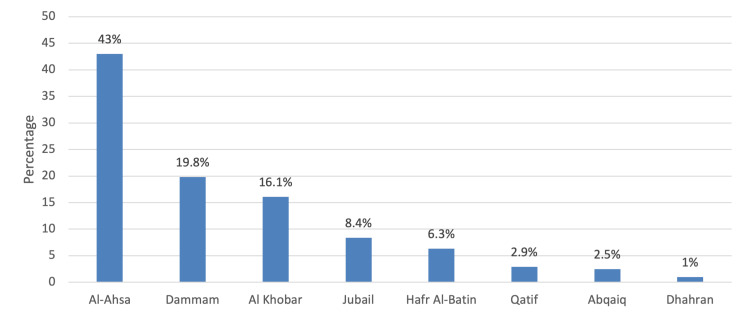
Location of residence in the Eastern province

In total, 688 participants responded to our survey (male 57.1% vs female 42.9%). Table 1 presents the sociodemographic characteristics of the children and their patients. Most children (64.5%) were in the younger age group (6-12 years). Forty-point-six percent (40.6%) of the respondents who filled out the questionnaire were brothers or sisters of the children. Regarding fathers’ education, more than half were bachelor’s degrees (52.8%) while mothers who were bachelor’s degrees were also of a similar proportion (54.4%). Regarding monthly income, 30.1% earned more than 10,000 to 20,000 SAR per month. Family consanguinity was reported by 43.2%. The most common type of residence was an apartment (53.1%). Approximately 56% of the respondents were living with four to six persons. The prevalence of parents who were suffering from obesity was 41.7%. Most children had a normal BMI (80.1%); however, 10.2% were classified as overweight and 5.1% were obese. Regarding residence location, 43% lived in Al Ahsa and 19.8% lived in Dammam (Figure 1).

**Table 1:** Sociodemographic characteristics of children and their parents (n=688)

Study Data	N (%)
Child age group	
6 – 12 years	444 (64.5%)
13 – 16 years	244 (35.5%)
Child gender	
Male	393 (57.1%)
Female	295 (42.9%)
Respondent's relation to the child	
Father/Mother	241 (35.0%)
Brother/Sister	279 (40.6%)
Other relatives	168 (24.4%)
Father educational level	
Below high school	44 (06.4%)
High school	173 (25.1%)
Bachelor	363 (52.8%)
Postgraduate	108 (15.7%)
Mother educational level	
Below high school	52 (07.6%)
High school	190 (27.6%)
Bachelor	374 (54.4%)
Postgraduate	72 (10.5%)
Family monthly income (SAR)	
<5,000	38 (05.5%)
5,000 – 10,000	116 (16.9%)
10,001 – 20,000	207 (30.1%)
20,001 – 30,000	200 (29.1%)
>30,000	127 (18.5%)
Consanguinity between	
Yes	297 (43.2%)
No	391 (56.8%)
Residence type	
Castle	21 (03.1%)
Villa	365 (53.1%)
Apartment	262 (38.1%)
Traditional home	40 (05.8%)
Number of family members	
3 persons	63 (09.2%)
4 – 6 persons	385 (56.0%)
7 – 9 persons	198 (28.8%)
≥10 persons	42 (06.1%)
Parents suffering from obesity	
Yes	287 (41.7%)
No	401 (58.3%)
Child BMI level	
Underweight (<5^th^ percentile)	32 (04.7%)
Normal (5^th^ – <85^th^ percentile)	551 (80.1%)
Overweight (85^th^ - <95^th^ percentile	70 (10.2%)
Obese (≥95^th^ percentile)	35 (05.1%)

Regarding children's characteristics and associated symptoms, 37.9% ate main meals three times a day (Table 2). The proportion of children watching electronic gadgets for more than two hours per day was 77.2% while the proportion of children who were physically active for more than two hours per day was only 10%. Multiple response answers indicated that the most common associated symptoms during the past year were stomachache (13.8%) and bloating (10.6%). Of those who complained about diarrhea (n=25), 52% defecated two to three times per day. Of those who complained about constipation (n=48), 54.2% defecated two to three times a day. Of those who complained about stomachache (n=95), 45.3% complained at least two to three times per month, whereas, of those who complained of vomiting (n=25), 44% did so two to three times per month.

**Table 2:** Children’s characteristics and associated symptoms (n=688)

Variables	N (%)
Child number of main meals per day	
One meal	28 (04.1%)
Two meals	196 (28.5%)
Three meals	261 (37.9%)
Four or more meals	203 (29.5%)
Number of hours watching electronic gadget	
Less than 30 minutes a day	18 (02.6%)
30 minutes to one hour a day	23 (03.3%)
One to two hours a day	116 (16.9%)
More than two hours a day	531 (77.2%)
Number of hours does the child spend doing physical activities	
Less than 30 minutes a day	302 (43.9%)
30 minutes to one hour a day	165 (24.0%)
One to two hours a day	152 (22.1%)
More than two hours a day	69 (10.0%)
Child-associated symptoms during the past year *	
Loss of appetite	63 (09.2%)
Bloating	73 (10.6%)
Diarrhea	25 (03.6%)
Constipation	48 (07.0%)
Stomachache	95 (13.8%)
Vomiting	25 (03.6%)
None	489 (71.1%)
Number of defecating per day of the child who complained about diarrhea ^(n=25)^	
Once	04 (16.0%)
2 to 3 times	13 (52.0%)
4 to 5 times	04 (16.0%)
More than 5 times	04 (16.0%)
Number of defecating per week of the child who complained about constipation ^(n=48)^	
Once	12 (25.0%)
2 to 3 times	26 (54.2%)
4 to 5 times	09 (18.8%)
More than 5 times	01 (02.1%)
Number of times complained about stomachache per month ^(n=95)^	
Once	20 (21.1%)
2 to 3 times	43 (45.3%)
4 to 5 times	19 (20.0%)
More than 5 times	13 ( 13.7%)
Once	
Number of times complained about vomiting per month ^(n=25)^	
Once	04 (16.0%)
2 to 3 times	11 (44.0%)
4 to 5 times	01 (04.0%)
More than 5 times	09 (36.0%)

**Figure 2:**
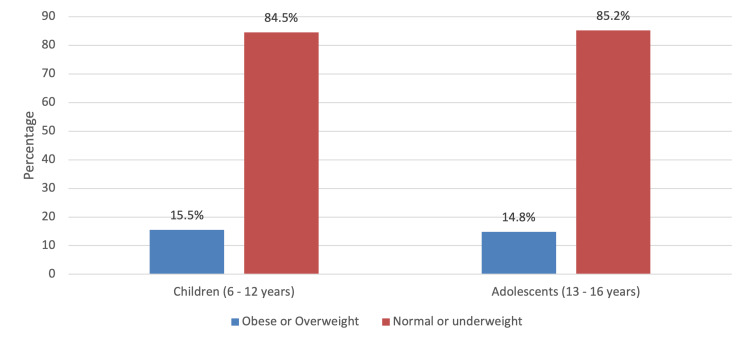
Level of BMI according to age group

**Figure 3:**
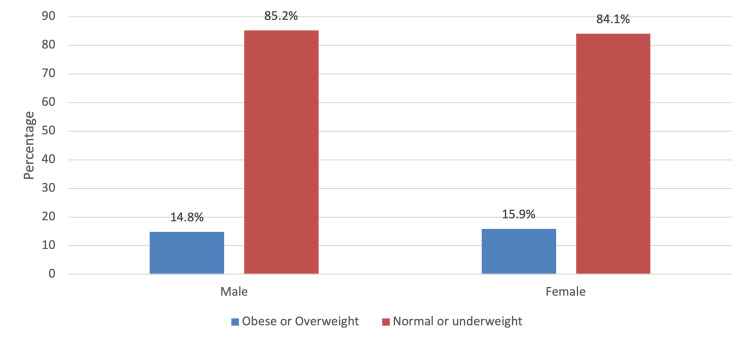
Level of BMI according to gender

When measuring the association between the BMI level and the socio-demographic characteristics of the children, it was found that there was a positive association between the BMI level according to consanguinity (p=0.038), number of family members (p=0.002), parents suffering from obesity (p=0.002), number of main meals per day (p<0.001) and number of hours spent in physical activity (p=0.009) (Table 3). Other variables included in the test did not show significant association with the level of BMI, including child age group, gender, family monthly income, number of hours watching electronic gadgets, and associated symptoms (p>0.05). In Figure 2, children were slightly higher on being obese (15.5%) than adolescents (14.8%) with no significant difference (p=0.784). In Figure 3, obesity or overweight was higher in females than males, but no significant difference was observed (p=0.672).

**Table 3:** Association between child obesity and sociodemographic characteristics of children (n=688) § P-value has been calculated using the chi-square test. ** Significant at the p<0.05 level.

Factor	BMI Level		P-value ^§^
	Obese or Overweight N (%) ^(n=105)^	Normal or Underweight N (%) ^(n=583)^	
Child age group			
6 – 12 years	69 (65.7%)	375 (64.3%)	0.784
13 – 16 years	36 (34.3%)	208 (35.7%)	
Child gender			
Male	58 (55.2%)	335 (57.5%)	0.672
Female	47 (44.8%)	248 (42.5%)	
Family monthly income (SAR)			
≤20,000	54 (51.4%)	307 (52.7%)	0.816
>20,000	51 (48.6%)	276 (47.3%)	
Consanguinity between			
Yes	55 (52.4%)	242 (41.5%)	0.038 **
No	50 (47.6%)	341 (58.5%)	
Number of family members			
3 persons	17 (16.2%)	46 (07.9%)	0.002 **
4 – 6 persons	48 (45.7%)	337 (57.8%)	
7 – 9 persons	28 (26.7%)	170 (29.2%)	
≥10 persons	12 (11.4%)	30 (05.1%)	
Parents suffering from obesity			
Yes	58 (55.2%)	229 (39.3%)	0.002 **
No	47 (44.8%)	354 (60.7%)	
Number of main meals per day			
One to two meals	23 (21.9%)	201 (34.5%)	<0.001 **
Three meals	27 (25.7%)	234 (40.1%)	
Four or more meals	55 (52.4%)	148 (25.4%)	
Number of hours watching electronic gadget			
Two hours or less a day	28 (26.7%)	129 (22.1%)	0.308
More than two hours a day	77 (73.3%)	454 (77.9%)	
Number of hours spent in physical activities			
Less than 30 minutes a day	43 (41.0%)	259 (44.4%)	0.009 **
30 minutes to one hour a day	16 (15.2%)	149 (25.6%)	
More than one hour a day	46 (43.8%)	175 (30.0%)	
Associated symptoms during the past year			
Yes	26 (24.8%)	173 (29.7%)	0.307
No	79 (75.2%)	410 (70.3%)	

When conducting a multivariate regression model, we observed that eating three or more main meals per day and physical activities for more than one hour per day were the significant independent factors associated with overweight or obesity (Table 4). This further suggests that compared to children who eat one to two meals per day, the odds of being obese or overweight for children who eat three meals per day could likely increase by at least 2.7 times higher (AOR=1.565 - 4.731; p<0.001) and 2.6 times higher among children who eat for or more meals per day (AOR=2.613; 95% CI=1.545 - 4.419; p<0.001). On the other hand, compared to children who were spending less than 30 minutes of physical exercise per day, the odds of obese or overweight for children who were doing physical exercise for more than one hour per day could likely decrease by at least 50% (AOR=0.476; 95% CI=0.253 - 0.894; p=0.021). Other variables included in the model did not show a significant effect on obesity or overweight after adjustments to the regression model, including consanguinity, the number of family members, and parents suffering from obesity (p>0.05). 

**Table 4:** Multivariate regression analysis to ascertain the influence of overweight/obesity from the selected socio-demographic characteristics of the children (n=688) AOR – adjusted odds ratio; CI – confidence interval ** Significant at the p<0.05 level

Factor	AOR	95% CI	P-value
Consanguinity between			
Yes	1.428	0.921 – 2.214	0.111
No	Ref		
Number of family members			
3 persons	Ref		
4 – 6 persons	0.863	0.345 – 2.159	0.753
7 – 9 persons	2.103	0.970 – 4.560	0.060
≥10 persons	1.898	0.836 – 4.307	0.125
Parents suffering from obesity			
Yes	1.444	0.921 – 2.263	0.109
No	Ref		
Number of main meals per day			
One to two meals	Ref		
Three meals	2.721	1.565 – 4.731	<0.001 **
Four or more meals	2.613	1.545 – 4.419	<0.001 **
Number of hours spent in physical activities			
Less than 30 minutes a day	Ref		
30 minutes to one hour a day	0.621	0.330 – 1.167	0.139
More than one hour a day	0.476	0.253 – 0.894	0.021 **

## Discussion

This study was carried out to evaluate the prevalence of obesity among children and adolescents in the Eastern province of Saudi Arabia and determine potential contributing factors to obesity in this population. BMI, which has been widely used as a measure of overweight and obesity, was adopted in this study. We found that 15.3% of the respondents were obese or overweight. This finding is consistent with Al-Hussaini et al., whose study reported an overall incidence of being overweight and obese at 13.4% among children living in Riyadh, Saudi Arabia [2]. Another study found a similar prevalence among Omani children [7]. However, some other studies have contradicted these reports. Athanasopoulos et al. documented a higher prevalence (28.9%) of overweight and obese children and adolescents on the island of Kalymnos, Greece [12]. Furthermore, the rates of obesity in Iran and the UAE have been reported as 28.2% and 29.1%, respectively [5,14]. Awareness about the increasing trend of BMI among school-aged children is necessary to address this alarming public health issue.

In the present study, obesity was more prevalent in children (15.5%) than in adolescents (14.8%) and among girls (15.9%) than among boys (14.8%). A similar sex-based trend was detected among children in South Africa [15]. The study accounts that using BMI cutoff points, the prevalence of obesity was higher in females than males. On the contrary, studies conducted in the USA and Serbia have reported a higher prevalence of obesity among boys than among girls [9,16].

In a study conducted in Nepal, being overweight and obese was linked to the types of food that individuals consumed regularly. Researchers noted that students who consumed energy-dense foods with fewer nutrients and students who were less active were more likely to be obese or overweight [6]. Among girls in Hail, Saudi Arabia, a significant correlation was found between obesity and the consumption of chocolates, sweets, soft drinks, and fast food [17]. In the present study, we found that higher daily food consumption was independently associated with overweight and obesity; eating three meals per day increased the odds of obesity by a factor of 2.7 while eating four or more meals per day increased those odds by a factor of 2.6. However, a study published in Oman found no significant association between BMI and the nutrients intakes of Omani children, which did not coincide with our reports [7].

Regular exercise is likely one of the best preventive factors against obesity, and our findings support this theory. Our data showed that more than one hour of physical activity per day among children and adolescents decreased the risk of obesity by at least 52%. A previous study conducted among Iranian primary-school children found that for every increase in the leisure-time physical activity score, the age-adjusted odds ratio of BMI decreased significantly [14]. Similar results have been reported in several studies from other countries [18-20]. Regular exercise is proven to be an effective measure of reducing weight. Therefore, parents must find creative ways to engage children/adolescents in physical activities, such as sports, to prevent sedentary lifestyles.

A previous study published in the USA stated that parental obesity was a predictor of excess weight in children. The study further explained that when a parent was obese, the risk ratio for childhood obesity was higher than 2.5. This indicated that when a parent had a BMI of 40 or higher, their child was five times more likely to be obese compared to a child of parents with normal BMIs [11]. In a study conducted on a Greek island, childhood obesity was linked to maternal obesity and maternal occupation [12]. Our study found that childhood obesity was associated with parental obesity and consanguinity, supporting previous reports.

Our study's increased prevalence of overweight and obesity may be related to the respondents’ daily routines. Approximately 30% of our study population consumed four or more meals daily, and more than 77% used electronic devices for more than two hours daily. These habits may mean less time spent doing physical activities. These observations are similar to those from Karki et al., whose investigation found that high junk consumption and sedentary activity were significantly associated with childhood overweight/obesity [6]. Similarly, Abduelkarem et al. reported that almost one quarter (23.9%) of school-aged children were physically inactive; they also reported significant rates of candy and fast food consumption (54.6% and 47.8%, respectively) [5]. There is a great need for school health and awareness programs aimed at reducing the consumption of energy-dense foods and promoting active lifestyles that include physical exercise.

## Conclusions

The prevalence of being overweight and obese was quite common. Increased daily food consumption was the risk factor for obesity while increasing physical activity was the protective factor against obesity in children and adolescents. Overweight/obesity in children and adolescents has become a significant public health issue in Saudi Arabia. Therefore, a healthy lifestyle based on effective dietary education and physical activity promotion is required to prevent overweight and obesity among youth. Hence, health planners and administrators should create appropriate strategies for children and adolescent obesity management.
